# The agency of fertility plans

**DOI:** 10.3389/fsoc.2022.923756

**Published:** 2022-11-25

**Authors:** Giacomo Bazzani, Daniele Vignoli

**Affiliations:** ^1^Department of Political and Social Sciences, University of Florence, Firenze, Italy; ^2^Department of Statistics, Computer Science, Applications, University of Florence, Firenze, Italy

**Keywords:** agency, capability approach, conversion factors, fertility, future, life-course, open questions, positive sociology

## Abstract

Fertility plans are a prominent area for agency research, and are a clear example of a misalignment between resources and agency capacity. We relied both on the idea of conversion factors of the Capability Approach and the pragmatist tradition of temporal-oriented agency to propose a framework for the study of fertility agency as the conversion process of resources into plans and behavior. We outlined said framework by using a unique dataset on fertility plans composed of open and closed questions from an Italian sample. Economic factors and imaginaries related to children and family represented the vast majority of (hindering and enabling) conversion factors. The notion of conversion factors is crucial for disentangling the network of heterogeneous elements involved in fertility agency: it allows focus to be shifted from structural factors related to social position and psychological characteristics to more situated elements that enable agency capacity.

## Introduction

As a prominent example of agency, fertility plans ought to receive greater attention than they have thus far been given in agency research. Indeed, the widespread use of the concept of agency focuses on the human capacity to be the “perpetrator” of the course of action (Giddens, 1984, p. 9).^1^In the modern contraceptive regime, childbearing tends to be intentional (Ajzen and Klobas, 2013) and remains a crucial part of life-course plans. Fertility plans are life-changing decisions that often require great effort in terms of planning and achievement. However, fertility's centrality on life-courses is not reflected in agency research. Indeed, despite empirical research on agency having mostly developed within the sphere of life-course research (Elder, 1994; Hitlin and Kwon, 2016), fertility plans and realizations have been largely ignored.

Fertility plans are a turning point in life trajectories. While many decisions have long-lasting consequences to one's life course (e.g., those related to education, migration, housing, etc.), childbearing decisions embody a definitive and irrevocable commitment to a long series of costs, burdens, and sleepless nights – but also expected happiness. Accordingly, childbearing decisions cannot fairly be compared with other choices.

In contemporary societies, the freedom of choice in several life-course domains has become normative (Giddens, 1984), and many decisions appear as (easily) reversible, such as those regarding marriage, residence, and labor. This is not the case for children, because “ex-children are a reality, but psychological and economic abandonment are not normative” (Morgan and King, 2001, p. 13). Children may well be the last frontier of individual freedom and the reversibility of choices; while everything is perceived as potentially reversible, “once the child arrives, there is no recourse to a resale market nor to a local humane society” (Turchi, 1975, p. 44). The choice to have children cannot be changed, and requires enormous levels of individual agency because of their impact on one's career, housing, and partnership decisions (Huinink and Kohli, 2014). The achievement of fertility agency requires both extensive planning and adherence to a subsequent set of behaviors. Fertility plans are the first outcome of fertility agency that require one to (re)consider a large part of one's personal life-course project according to the expected needs of children. In this sense, fertility plans are a unique space of “hyperprojectivity” of future life-courses with the ability to shape present decisions and require a high standard of projective agency. As such, fertility agency should be more comprehensively explored within agency research.

Empirical research on life-course agency has typically foregrounded the analysis of the socio-psychological characteristics able to account for the heterogeneity of individual success across the life-course (Elder, 1994; Hitlin and Kirkpatrick Johnson, 2015; Hitlin and Kwon, 2016). However, agency capacity is often not a direct consequence of the availability of structural and personal resources, but rather heavily influenced (both positively and negatively) by many situational conversion factors (Hobson, 2018; Hvinden and Halvorsen, 2018; Choi et al., 2019). For instance, the effect of economic uncertainty on fertility plans may be twofold. On the one hand, uncertain economic conditions may suggest avoiding such long-term binding decisions as fertility (Mills and Blossfeld, 2013); whereas on the other, they may also incentivise devaluing career prospects in favor of fertility plans (Friedman et al., 1994).

We here study fertility agency based on recent sociological developments on Sen's Capability Approach (Kremakova, 2013; Gangas, 2016; Hobson, 2018; Hvinden and Halvorsen, 2018; Zimmermann, 2018; Bazzani, 2022; Hobson and Zimmermann, 2022), hereafter CA. Sen usefully distinguishes between the “capability inputs” – to refer to the entire set of personal and structural resources – and two types of agency: “freedom” and “achievement.”^2^ This distinction suggests focusing on the disconnect between a generic desire to have children and available resources (capability inputs) on the one hand, and the actual fertility plans (agency freedom) and consequent realization (agency achievements) on the other. Interestingly, even in structurally favorable conditions, personal resources are not necessarily *converted* into actual fertility plans and behavior. Sen has emphasized that capabilities and achievements vary depending on the institutional, societal, and cultural context, as well as on the research question. In the CA, the factors that convert resources into agency are defined as *conversion factors* (Sen, 1992; Robeyns, 2017). The study of the conversion factors of projectivity is a new frontier for research. Hobson and Zimmermann (2022) provide a framework with a multi-dimensional concept of agency that reveals the pathways from projectivity and the capability to aspire to the potential for choice (freedoms) that lead toward achieving goals.

Fertility trends in wealthy countries aptly demonstrate unachieved agency capacity as they show an increasing misalignment between desire, actual intentions, and behavior (Hagewen and Morgan, 2005). People reserve resources with which to plan childbearing – in terms of reproductive capacity, economic resources, and housing conditions – but they frequently opt to postpone or avoid it entirely. Research has primarily addressed the individual socio-economic circumstances and welfare regimes to explain unachieved fertility (Balbo et al., 2013), but the future orientations of the deliberative process that is part of fertility agency has often been underestimated (Vignoli et al., 2020a). The majority of relevant research has had no direct access to the “voices” that could help disentangle the relational nature of fertility agency – although there are notable exceptions to this (e.g., Randall and Koppenhaver, 2004; Bernardi et al., 2008; Mynarska, 2010; Brinton et al., 2018; Lebano and Jamieson, 2020). In this sense, fertility agency remains a black box that cannot be reduced to structural factors (e.g., social class, family policies; Balbo et al., 2013) or psychological characteristics (e.g., sense of mastery, risk aversion; Bellani and Arpino, 2021). Indeed, “very little is known from a sociological perspective about how and why people make decisions in the present about their lives [...] self-conceptions like self-efficacy undoubtedly matter, but their exact links to decision-making have not been established” (Shanahan and Macmillan, 2008, p. 214).

This article makes two primary contributions to the field, one theoretical and one empirical. First, we propose a framework for the study of the *conversion processes* and the *conversion factors* of fertility agency based on the recent sociological development on Sen's CA. Second, we offer an initial application of this framework by using a unique dataset based on an Italian sample of respondents (*N* = 276). Our data includes both open and closed questions which we examined through a qualitative analysis augmented with frequency distributions across different groups of respondents. We have focused on fertility plans as the first step of fertility agency. The analysis considered the hindering and enabling conversion factors involved in the fertility decision-making process. Unlike most related studies, we used an inductive approach (Boyatzis, 1998) to identify the relevant factors without assuming a predetermined set of variables.

## The conversion processes of fertility plans

### Fertility plans: A space of hyperprojectivity

Agency embodies three types of temporalities: iteration, projectivity, and practical evaluation (Emirbayer and Mische, 1998). “Iteration” refers to actors' routine reactivation of past patterns of thought and action, “projection” entails the capacity to imagine possible future action trajectories, and “practical evaluation” is the capacity to make practical and normative judgments based on present alternatives and deliberations (Emirbayer and Mische, 1998, p. 917).^3^ Fertility plans are long-term in nature, where routine tends to gives way to the agency of projectivity and deliberation (Mische, 2009). Child-bearing is a life-changing decision that requires one to first imagine the future condition of parenthood (projective agency), then a choice on whether and when childbearing should occur (practice evaluative agency). In a modern contraceptive regime, childbearing tends to be intentional (Ajzen and Klobas, 2013), though not in the form of rational cost-benefit calculations. Fertility plans are truly uncertain due to the unforeseeable nature of the long-term consequences of planning or postponing childbearing (Vignoli et al., 2020a). This is because such decisions are always taken in conditions of uncertainty (which is not to say risk), and they are guided by a narrative of the future that can be more or less plausible and normatively oriented (Bazzani, forthcoming; Tavory and Eliasoph, 2013; Tuckett and Nikolic, 2017).^4^

Fertility plans “force” people to de-routinise their courses of action. Routine can be interrupted by the emergence of conflict between “different habits, or by the release of impulses” (Beckert, 2016, p. 54),^5^ wherein individuals experience uncertainty over the future that require (new) judgments, thereby allowing the deliberative process to emerge. In an uncertain situation, past experiences and expectations become relevant in an imaginative “dialogue” considering “competing possible lines of action” (Dewey, 1930, p. 190).

In the routine condition, different possible narratives of the future life-course may co-exist in a vague or undecided scenario. Parental pressure for grandchildren, peers' parenthood experiences, and personal imaginaries of children may interrupt young adults' routines and force them to problematise their daily lives, thus causing them to consider childbearing when they would not otherwise have done so. Fertility decisions force people to re-shape future life plans and establish new narrative of the future, including even postponing or abandoning fertility plans. In this sense, fertility plans are a personal space of “hyperprojectivity.” Mische (2014) developed the concept of hyperprojectivity by studying arenas of heightened, future-oriented public debate about contending futures, such as those occurring in communities, social movements, and policy arenas. We use this concept to describe a similarly high level of projective capacity taking place at the personal level of fertility plans: considering an imagined long-term future forces one to reconsider the present situation based on its strengths and weaknesses, thus potentially leading to new plans. The level of household income or expected employment stability are often considered prerequisites to parenthood (Alderotti et al., 2021). However, the specific characteristics of such prerequisites are always contingent and re-evaluated, such as in view of personal age (micro-level), the quality of the relationship with the partner (meso-level), or the availability of family policies (macro-level).

### Enabling and hindering conversion factors

Fertility plans are the outcome of the imaginative dialogue on life-course outcomes. However, these plans cannot be reduced to either personal will or psychological forces that are the common factors operationalised in agency research. These factors include self-esteem (Cast and Burke, 2002), self-efficacy (Bandura, 1986), mastery (Pearlin and Schooler, 1978), personal control (Mirowsky and Ross, 2007), optimism for the future (Frye, 2012), and planful competence (Clausen, 1991). Indeed, while “emotions and personality traits – along with idiosyncratic personal histories, moral codes, and predispositions – influence the choices we make in emergent situations” (Hitlin and Elder, 2007, p. 178), there are also contingent situational contexts that influence courses of action. From a sociological perspective, it is crucial to understand “what kinds of contexts provoke or facilitate them [actors] toward gaining imaginative distance from those responses and thereby reformulating past patterns through the projection of alternative future trajectories” (Emirbayer and Mische, 1998, p. 1006). To this end, Hobson suggested that “situated agency is a more sociological concept capturing the relational aspects of agency, the diversity in individual situations that shape agency freedom, and the potential to convert resources into achievements” (2018:11). The concept of situated agency (Peter, 2003; Choi et al., 2019) is useful for considering the relational dimension of agency (Abbott, 2020), in that it is “always agency toward something, by means of which actors enter into a relationship with surrounding persons, places, meanings, and events” (Emirbayer and Mische, 1998, p. 973).

From this perspective, fertility plans are not only the direct effect of some pre-existing internal anthropological force (e.g., desire or impulse), but they are also oriented by contingent factors that shape the course of action.^6^ Personal resources (e.g., a stable partnership and the desire for children) are frequently unconverted into fertility plans and behavior due to situational hindering factors. Indeed, agency freedoms, involve more than “desire,” but embrace agency and choice and the power to act on choices (Hobson and Zimmermann, 2022). The misalignment between resources and desire (capability inputs), and actual plans and realization (agency freedom and achievements), seems to suggest the utility of considering the interplay between situational *conversion factors* and the long-term processes, such as the individual characteristics and structural factors explored within the literature. Indeed, this gap between desires and actual fertility plans may stem from a wide array of factors, as “unresolved tensions between choice and freedom and puzzling paradoxes mark individual agency” (Lebano and Jamieson, 2020, p. 125). Within the capability framework, conversion factors can determine whether or not individual resources and structural factors are converted into agency through a *conversion process*.

**Figure 1:**
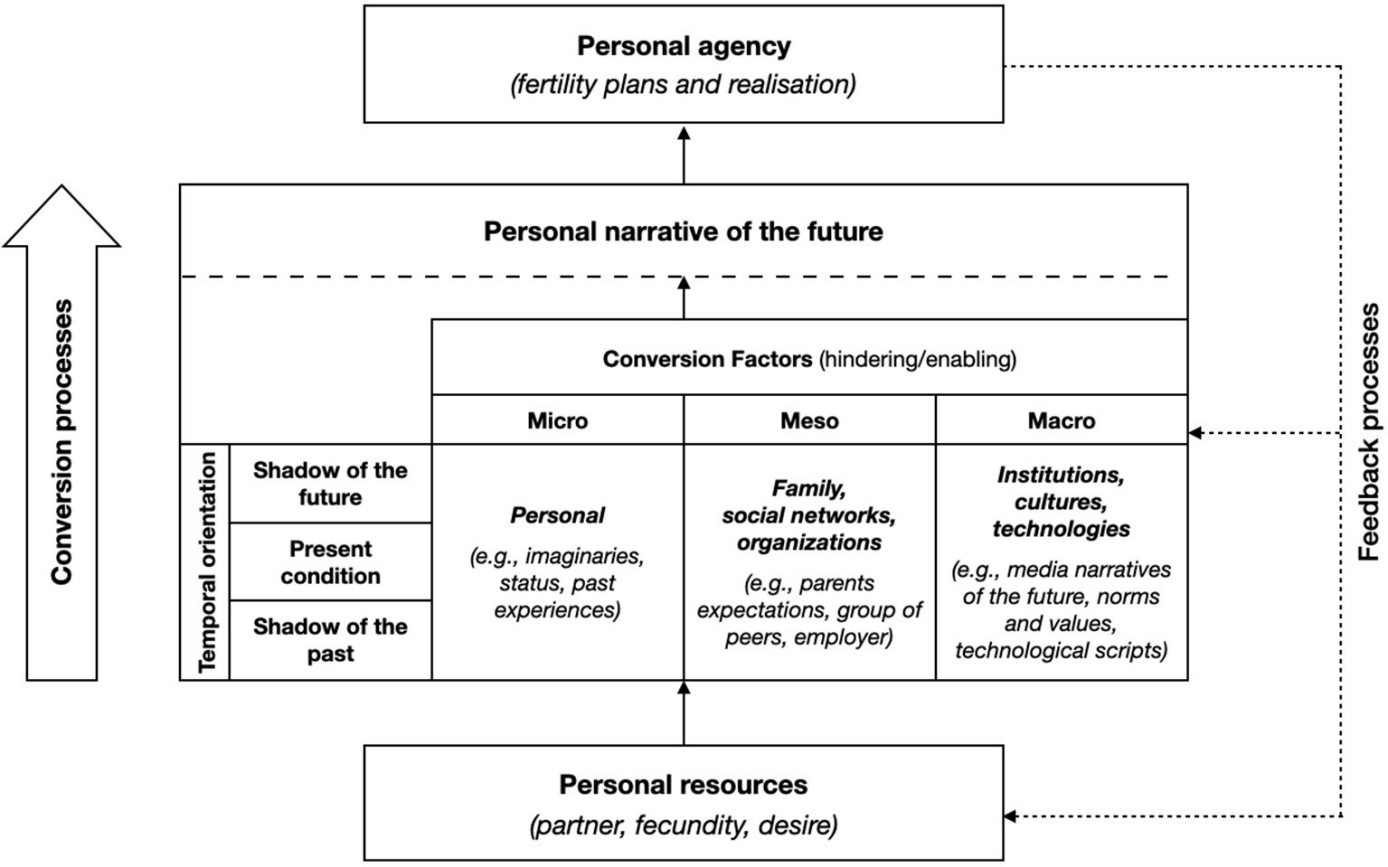
Fertility conversion processes (adapted from Bazzani, 2022).

Recent developments have translated into a more sociological formulation of Sen's conversion factors that convert resources into agency achievement Sen (1999, 2005, 2008), considering the micro-, meso-, and macro-levels of analysis, as well as feedback processes (Hvinden and Halvorsen, 2018; Bazzani, 2022). Hobson and Zimmermann's (2022) model of future making involves a multi-dimensional relational dynamic of situated agency which can be enabled by the capability to aspire and activated through the conversion of institutional, organizational, cultural and societal resources. In our multi-dimensional framework, the three types of temporalities elaborated by Emirbayer and Mische (1998) are considered together with micro, meso, and macro level conversion factors that enable or hinder fertility agency. Figure 1 illustrates a stylised representation of the conversion processes of fertility agency with the different levels and elements involved (conversion factors), including their temporal orientation.^7^ The schema accords with the idea of multi-level structures influencing fertility and other life-course dynamics (Matysiak and Vignoli, 2010; Huinink and Kohli, 2014; Billari, 2015; Bernardi et al., 2019).

The iterative dimension of fertility agency is embedded in the elements belonging to the “shadow of the past” and the “present condition,” despite often deploying their effects on future expectations. Projectivity agency is influenced by the “shadow of the future” and synthesized in the “personal narrative of the future,” which also includes fertility decisions (practical evaluative agency; Emirbayer and Mische, 1998; Mische, 2009, 2014). The conversion processes mediate the relationship between personal resources (e.g., stable relationship, fecundity) with agency (fertility plans and behavior), and contain several elements that may be involved as conversion factors. The conversion factors are presented as a typology organized according to their level (micro, meso, and macro) and temporal orientation (past, present, and future)^8^ (Bernardi et al., 2019). Moreover, conversion factors could either “hinder” or “enable” the process. Some of the conversion factors that may influence fertility plans have already been considered within the literature. The following section introduces the most influential theories for explaining the fertility declines observed in wealthier countries, and discusses their major shortcomings.

## Main perspectives on low fertility

According to the Second Demographic Transition (SDT) theory – perhaps the most influential theory on fertility dynamics developed in post-war Europe – individuals (and secularized women in particular) reprioritise their own careers and self-actualisation over family and childbearing (Van de Kaa, 1987; Lesthaeghe, 2020). This long-term cultural change has been observed in most wealthy countries, albeit to different degrees of intensity. However, SDT does not explain the persistence of the two-children ideal in the context of widespread post-modern values. Moreover, the simple opposition between self-fulfillment and traditional family values does not effectively capture the complex meanings and family imaginaries associated with fertility plans in contemporary societies. Indeed, the loss of children's economic value in wealthy countries has not been associated with a parallel loss of “any” value of children (Zelizer, 1985).

Psychological cross-cultural studies on the value of children have shown that, in wealthy countries, the utilitarian-normative values of children have been replaced by their emotional value (Trommsdorff and Nauck, 2010; Nauck, 2014) – which is also occasionally connected with religious orientation (Adserà, 2006; Peri-Rotem, 2016; Dilmaghani, 2019). Religious affiliation could be a conversion factor of fertility intentions capable of providing a normative orientation in a vague or undecided future scenario (Philipov and Berghammer, 2007; Bein et al., 2021). For instance, beliefs about the “sanctity” of the family or ultimate life goals may offer a clear future narrative to orient one's personal efforts in the present and resolve uncertainties about the role of children in one's life-course. Such beliefs emphasize the emotional value of children as “priceless” (Zelizer, 1985), and the requirement to heavily invest in them. Paradoxically, the individualization process may have created a model of parenthood known as “intensive parenting” (Hays, 1996; Gauthier et al., 2020) or “total motherhood” (Lebano and Jamieson, 2020), which seems highly incompatible with career development. Some studies have considered the driving role of children imaginaries in fertility plans, with a special emphasis on the imagined type of care relationship (Lebano and Jamieson, 2020; Gauthier and De Jong, 2021). However, research on family imaginaries associated with parenthood remains relatively scant, thus highlighting the need for a greater focus on the sources of aspiration or disinterest for children.

A different stream of research has instead focused on mounting (economic) uncertainty as a consequence of globalization and neoliberal policies (McDonald, 2006). Economic crises and social transformations associated with globalization and neoliberal policies act as constraints on intended and actual fertility (Mills and Blossfeld, 2013). At the micro-level, time-limited employment often reflects a low level of labor-market integration, which is typically associated with weak employment protection and wage penalties, thus generating feelings of uncertainty. This condition seems incompatible with such long-term binding decisions as fertility plans (Schmitt, 2012; Mills and Blossfeld, 2013). Moreover, according to the “relative income hypothesis,” this constraint effect is emphasized because people tend to evaluate the suitability of their income for childbearing according to the living standards of the family of origin (Easterlin, 1980).

However, objective economic indicators alone are perhaps not the most suitable proxies of perceived economic conditions. The Narrative Framework is a recent perspective that suggests considering expectations and imaginaries as crucial elements of the narrative of the future which can influence fertility plans (Vignoli et al., 2020a,b). Empirical support for the mounting importance of future expectations and imaginaries for fertility plans has been recently proffered (e.g., Gatta et al., 2021; Vignoli et al., 2022), especially in light of the COVID-19 pandemic (Guetto et al., 2022; Manning et al., 2022). Interestingly, a high level of economic insecurity may both hinder or enable fertility conversion factors (Friedman et al., 1994). For instance, for lowly-educated women, motherhood may be a route to building personal identity and thus help stabilize future life prospects (Kreyenfeld, 2010). However, qualitative research on perceived economic factors and their salience in personal narratives remains limited (see Bernardi et al., 2008; Mynarska, 2010; Brinton et al., 2018; Bueno and Brinton, 2019; Lebano and Jamieson, 2020), especially for identifying the twofold role of hindering and enabling factors on fertility plans.

## The current study and its context

The current study is an initial application of the proposed framework that explores the conversion factors which hinder or enable fertility intentions in the Italian context. Since 2010, total fertility rates (TFR) have dramatically declined in several European countries, dropping to far below the replacement level. In Southern Europe, Italy, after having witnessed a fertility rebound at the beginning of the new millennium, is now experiencing a constant fertility decline, officially re-entering the lowest-low category in 2019, with a TFR of 1.29 (ISTAT, 2019). In particular, we seek to detect: (1) which types of family and children imaginaries are mentioned in relation with fertility plans; and (2) which perceived economic factors related to fertility intentions are more salient to personal narratives of the future.

## Methodology

### Sample

The data employed in the analyses were taken from a survey organized by the University of Florence between June 2019 and early February 2020 – i.e., before the COVID-19 pandemic. The survey was part of a laboratory experiment dedicated to studying the influence of economic uncertainty on fertility intentions, and was planned by a multidisciplinary team of demographers, sociologists, social psychologists, and economists. The team met several times throughout the course of a year to design the questionnaire and run pilot tests. The survey protocol was approved by the Ethics Committee of the University of Florence and was then updated after the pilot, whereupon it was once again approved by the Ethics Committee. Please see Vignoli et al. (2022), Appendix 1 for further details.

The analyzed data came from a subsample of respondents consisting of the control group (1/3 of the sample) who were not exposed to any lab treatment and only answered the survey questions. Our participants consisted of 276 respondents aged between 20–40 in stable relationships (i.e., married, cohabiting, or living apart). We ensured a balanced participation by sex, as well as between the jobless, permanently employed, and temporarily employed. The participants were recruited through the services of a specialized survey agency, which gave no information as to the content of the research (i.e., no references to family or economic aspects were made). The agency was also asked to try to ensure a distribution by education and parity that would mimic that of the national population distribution, but without imposing strict quotas. Participants were selected from panel participants available to the agency, boosted with additional participants recruited through advertisements distributed in public places, again without providing any information about the experiment's content.

### Data

We asked the participants to answer a large array of demographic, socio-economic, and psychological questions. The first part of the questionnaire was dedicated to fertility intentions, followed by open questions about the role of uncertainty in their lives and the personal narratives associated with fertility intentions (Vignoli et al., 2022). They were asked about their fertility intentions for the following 3 years (Q: “Do you intend to have a child in the next 3 years”). We focused on fertility intentions as it is the first step of fertility agency and a good predictor of subsequent behaviors. Indeed, fertility intentions “in close temporal proximity to the prospective behavior” (Ajzen and Fishbein, 1973, p. 49) are generally considered suitable predictors of actual behavior (Philipov, 2009; Spéder and Kapitány, 2009; Régnier-Loilier et al., 2011). Following recommendations from the psychology literature, we assessed fertility intentions on a scale of 0 to 10 (where 0 corresponded to “definitely not” and 10 to “definitely yes”) in order to grasp individual differences in psychological constructs with acceptable levels of precision (MacCallum et al., 2002). This choice allowed us to address both the direction and intensity of fertility intentions. The intermediate point of the scale was also included so as to capture ambivalent or neutral positions (Zammuner, 1998). We implemented the survey using the O-TREE open-source platform (Chen et al., 2016).

We analyzed the answers to two open questions about personal narratives associated with fertility intentions. The first focused on personal aspects: “When you were asked if you intend to have a child in the next 3 years on a scale from 1 to 10, you answered X. Which aspects of your personal situation were more important in deciding your answer?” The second considered the role of macro-economic factors on fertility decisions: “When you were asked if you intend to have a child in the next 3 years on a scale from 1 to 10, you answered X. Which aspects of the economic situation of your country were more important in deciding your answer?”. We refined both questions following the results of successive pilot tests to ensure their clarity and capacity for capturing the information most relevant to the research aims. Instead of using two different questions for the micro and meso conversion factors, pilot tests showed that a single open question (on their “personal situation”) was able to encourage the respondents to consider both micro and meso aspects. This strategy allowed the respondents to separately consider the micro and meso aspects while also giving them the freedom to report on one or both aspects, thereby enabling us to understand the similarities and differences of their personal narratives. While in-person interviews on family planning may be oriented toward social desirability, social norms, and interviewer expectations, we attempted to overcome these biases by collecting the answers to the open-ended questions through an anonymous form completed on a computer (Colton and Covert, 2007).

Fertility intentions for the next 3 years are part of fertility plans that are influenced by – but do not necessarily coincide with – personal imaginaries related to children. Answers about abstract ideal family size are more likely to reflect societal norms than those about personal imaginaries of family and children (Sobotka and Beaujouan, 2014). Exploring how individuals depict their family imaginaries is further complicated by social desirability and cognitive dissonance biases (Burke and Stets, 1999). Following the literature on expected happiness (Billari, 2009; Mencarini et al., 2018) and affective forecast (Wilson and Gilbert, 2003), we asked the respondents (on a scale of 0 to 10) how much happier would having a(nother) child make them. Higher values can be interpreted as the ultimate outcome of a positive family imaginary that would eventually allow one to identify the gap between family imaginaries and real fertility intentions.

Drawing distinct measures between fertility intentions and the expected happiness that having a(nother) child could bring allowed us to more closely focus on analyzing the enabling and hindering conversion factors of fertility intentions within the respondents' personal narratives. Accordingly, the analysis explored two distinct subsamples of respondents. We analyzed the enabling factors in the answers to the open questions for respondents with fertility intentions from 5 to 10 (*Achieved capability group, N* = 146), and the hindering factors among the respondents with fertility intentions lower than the value attributed to the expected happiness of a(nother) child (*Unachieved capability group, N* = 166). We assumed that this group of respondents had faced some obstacles to their fertility intentions that we hoped to explore in their answers to the open questions. The two groups partly overlapped by construction: The achieved capability group was used to code the factors enabling fertility intentions, and the unachieved capability group to code the factors hindering it. The descriptive statistics of the sample and the two subsamples can be seen in Table A1 of the Supplementary material.

### Analytical strategy

Open question data (which has been typically unused in most previous studies in the field) has the advantage that the analyst has access to the respondents' ideas in their own words. This opens up possibilities of using content analysis to search for subtler patterns within the responses compared with closed questions (Fielding et al., 2013). In order to analyse these patterns, we used an inductive approach to identify the relevant factors without assuming a predetermined set of variables (Boyatzis, 1998). We analyzed the answers to the two open questions using MAXQDA-2020 software, and coded them according to the content analysis approach (Züll, 2016) for both the achieved and unachieved capability groups. We anonymised the answers prior to the analysis. In particular, the interview coding sought to capture the different conversion factors that people felt were relevant in their fertility decision-making processes. We used an iterative process that started with a round of open coding. All of the transcripts were read, whereupon we highlighted recurring themes and reflected on emergent issues in a series of memos. This process generated a list of initial codes that we then used in a second review of each transcript. Initial ideas and themes were refined in the context of this second review. Double coding of randomized subsamples of the materials was conducted by a second coder to assess the reliability of the coding procedures. Differences in coder agreement were minimal and resolved *via* discussion. Once done, the refinements involved both splitting categories into those with finer distinctions and combining categories as deeper themes became apparent (Fielding et al., 2013). In the next step, we grouped the different factors according to the varying micro, meso, and macro levels of the conversion factors (Figure 1). We interpreted the presence of the different self-reported factors related to fertility plans as a signal of their influencing capacity within the conversion process of fertility agency. Thus, as an application of the inductive method, we generated a table setting out the numbers or proportions of responses that referred to each of the conversion factors identified in the data (Sandelowski, 2001; Fielding et al., 2013).

The answers to the two open questions shed light on the personal narratives associated with fertility agency, which represent agency's projective and deliberative side (Emirbayer and Mische, 1998). However, there may well be conversion factors that are part of the iterative side of agency of which people are either simply unaware, or follow a “silent” reduction and production of possibilities (i.e., “manifest in automatic rather than deliberative cognition,” DiMaggio, 1997, p. 269–71). While these types of conversion factors are “structural” and influence the fertility decision-making process, they are not readily apparent when directly analyzing personal narratives. Indeed, people may be unaware of their influence or dismissive of their relevance. We conducted the analysis of the role of structural conversion factors on personal narratives with a convergent analysis with the data-based integration of answers to the open and closed questions (Fielding, 2012; Morgan, 2014; Kuckartz and Rädiker, 2019). Once the thematic coding had been completed and checked, we analyzed the distribution of the patterns of responses by using the code frequencies across different groups of respondents with cross-tabulation functions. This allowed us to generate tables based on selected combinations of thematic codes and attributes (‘quantitative analysis of code frequencies broken down by groups' Kuckartz and Rädiker, 2019, p. 179; Fielding et al., 2013; see also Sandelowski, 2001; Kuckartz, 2014, p. 140–142; Guetterman et al., 2015; Hesse-Biber et al., 2015, p. 5–7). We used the answers to closed questions on working conditions, education level, sex, religiosity, and the number of children to observe the different distribution of the conversion factors among those groups.^9^

To grasp the effects of unemployment or temporary job contracts on fertility plans, we classified the respondents according to their employment condition (1 = employed with a permanent contract; 2 = employed with a temporary contract; 3 = jobless). Regarding education's impact on fertility, we distinguished the respondents' education levels between low (1 = elementary, junior high school, and short vocational courses), medium (2 = high school), and high (3 = tertiary or higher). The respondents' religious affiliation was classified by distinguishing between those who felt themselves as belonging to a specific religion and those who did not. The overwhelming number of religious respondents (88%) declared themselves to be Catholics. We conducted the analysis for first-birth, second-birth, and higher-order parity separately, and divided the respondents between the childless and parents with one, two, and three children for the unachieved capability subsample. Due to their limited number, we did not consider respondents with two or three children (four and five respondents, respectively) as being part of the achieved capability subsample.

We interpreted the results of the analysis of the distribution of codes among different groups of respondents as the presence of different/similar conversion factors in personal narratives associated with fertility intentions (Kuckartz and Rädiker, 2019).^10^ In some cases, we observed unexpected results in the distribution patterns of the conversion factors among groups. In such cases, we developed qualitative insights on specific subsamples.

## Results

We first present the results of the hindering factors to fertility plans from the unachieved capability group, after which we turn to the enabling factors from the achieved capability group. The analysis separately considered the answers to the open questions on personal situations and the country's macro-economic situation.

### Personal hindering factors to fertility intentions

At the micro-level, the most frequently reported hindering factors were perceptions and expectations concerning the economic situation and imaginaries related to children and family (Table 1, column 1). Indeed, economic factors were reported in half of the coded segments. Among the hindering economic factors cited, the respondents mentioned precarious jobs, low-income levels, joblessness, as well as uncertainty and generic economic troubles. Precarious jobs refer to various types of working conditions, from seasonal work with temporary contracts (“Mainly my work situation, working seasonally I cannot ensure security in the first place for me, *in secundis* for my future children” female, 30) to self-employment with a relatively developed career. Uncertainty and generic economic troubles often refer to – without specifying an exact meaning – an “uncertain personal economic situation” or “employment situation.”

**Table 1:** Code frequencies of the hindering factors to fertility intentions in cases of the unachieved fertility capability.

**Hindering factors**	**Sample**		**Working condition**			**Level of education**			**Sex**		**Number of children**			
	**%**	***N***	**No work %**	**Temp %**	**Perm %**	**Low %**	**Med %**	**High %**	**F %**	**M %**	**0 %**	**1 %**	**2 %**	**3 %**
	1	2	3	4	5	6	7	8	9	10	11	12	13	14
**Micro**
**Perceptions, Expectations**
*Economic*	39.4	71	54.3	49	23.2	27.3	38.7	46	42.2	37.3	51.9	32.1	20.8	30
*Family and work conciliation*	11.8	20	13.0	11.8	10.1	4.5	11.8	14	13.3	9.6	12.7	7.5	8.3	30
*Housing*	12.4	21	10.9	13.7	13	9.1	12.9	12	10.8	14.5	19	9.4		10
*Age*	10	17	8.7	11.8	10.1	9.1	11.8	8	14.5	6	7.6	11.3	12.5	20
*Health*	6.7	11	8.7	3.9	7.2	9.1	7.5	4	6	7.2	2.5	9.4	16.7	
**Imaginaries**
*Desire of self-realization and freedom*	14.5	25	17.4	17.6	10.1	9.1	14	18	15.7	13.3	24.1	1.9	12.5	10
*Child requires responsibility care time*	6.7	11	8.7	3.9	7.2	9.1	6.5	6	3.6	9.6	6.3	7.5	8.3	
*Inadequate standard of living for children*	5	8	4.3	3.9	5.8	4.5	6.5	2	6	3.6	5.1	3.8	4.2	10
*Inadequate personal maturity*	2.5	4	2.2	3.9	1.4		1.1	6	2.4	2.4	3.8	1.9		
**Meso**
*Other Children*	21.8	36	19.6	15.7	27.5	22.7	22.6	20	24.1	19.3		24.5	62.5	80
*Partner characteristics and relationship*	11.2	19	4.3	11.8	14.5	4.5	14	8	7.2	14.5	15.2	5.7	8.3	10
*Partner negative intention and imaginaries*	5	8		7.8	5.8		4.3	8	3.6	6	5.1	3.8	8.3	
*Family of origin support*	6.7	11		9.8	8.7	4.5	7.5	6	7.2	6	6.3	1.9	20.8	
*Gender equality in the couple*	0.6	1	2.2					2	1.2		1.3			
**Macro**	10.6	18	8.7	11.8	11.6	18.2	10.8	6.0	8.4	13.3	12.7	9.5	12.5	
*N* respondents	166		46	51	69	22	93	50	83	83	79	53	24	10
% of the sample	100		27.7	30.7	41.6	13.3	56	30.1	50	50	47.6	31.9	14.5	6

Distribution among different working conditions, education level, sex, and parities. Sample: Unachieved capability group of respondents with fertility intentions lower than children imaginary (*N* = 166, uncoded document = 27; column % among the coded documents). The distribution between religious and irreligious respondents is not shown because it provided no specific difference.

Interestingly, the economic hindering factors were not only reported by the jobless or the temporarily employed, but also (albeit less frequently) by those with permanent work contracts (Table 1, column 5). Despite the absence of precarious employment, these respondents reported sharing a common feeling of progressive deterioration of working conditions. “The working environment is too precarious, the economic condition is very important to be able to guarantee a future for our children” (male, 43, permanently employed). Moreover, some stably-employed respondents reported low-income levels. “The situation in the world of work whereby workers' salaries do not follow the trend in the cost of living and the consequent increases in the cost of all accessory and essential goods” (male, 32, permanently employed).

The distribution of the economic hindering factors among different education levels followed an unexpected increasing pattern for highly-educated respondents (Table 1, columns 6, 7, and 8). While we may have expected that high education levels would correspond with better employment conditions (which, in turn, would not represent an obstacle to fertility intentions), the distribution pattern of economic factors suggests their salience also for the highly-educated subgroup. This is because high levels of education do not always correspond with better working conditions. “In the historical period in which we find ourselves, we young precarious people, with difficulties in creating a stable position, find it difficult to set ourselves such goals. We need to think more about surviving in the current world of work than we do about having a child” (male, 34, high education). Some respondents began professional careers which often require years of work before a stable income is granted. “I work, I am a practicing attorney and the path is very long if you want to go freelance. The earnings are very random” (female, 32, high education). Indeed, this low-income obstacle was also shared by respondents with jobs requiring high levels of education. “Bha, last year when I worked as an educator in a cooperative I earned 6 euros net per hour, how the fuck do you raise a child with a salary like that?” (male, 27, high education).

While it is obvious that the economic cost of children increases in line with their number, the reported salience of economic hindering factors for having another child, conversely, decreased after one child, and much more significantly after two (Table 1, columns 11, 12, 13, and 14). The types of reported concerns were similar across different parities and regarding both the precarious job condition and the low-income level (Table A2). Different hypotheses can be advanced for this unexpected phenomenon. For instance, we could speculate that families with one or two children were selected among those with greater economic resources. We calculated the mean monthly salary of the respondents, but found it to be relatively similar across the different groups, thereby not supporting the selection hypothesis.^11^ Alternatively, we may also hypothesize that childless people have unrealistic expectations of the cost of raising a child. The perceived capacity of coping with the economic and personal burdens of childrearing may increase with the parenthood experience together with the decreasing salience of the economic aspects. However, it seems reasonable to argue that the experience of parenthood changes the attributed salience of different aspects of people's lives, including the economic prerequisites. “With our first daughter, we were conditioned by our and our country's economic situation. We put it off for several years hoping for a better time, but since she is here, now 3 years old, there could not be a better time” (female, 34).

Among perceptions and expectations, inadequate housing conditions for hosting a(nother) child and hardships in work-family conciliation were often reported. Regarding the latter, people reported that they expected to be unable to successfully meet their children's needs or have sufficient time to work. Conciliation problems were associated both with the need for career development and the absence of flexibility in the working schedule. “I wish I had achieved a more stable and better paid position, lack of flexibility regarding working hours – I am not allowed to work remotely” (male, 31). In some cases, school timetables did not coincide with parents' working hours. “I already have two children, no help (not even financial) close other than my husband. The management is all in my hands. At the slightest hitch, there is a problem. School hours do not coincide with work hours. My contract was not renewed when it expired after returning from my second maternity leave” (female, 33).

Imaginaries related to children and family were frequently cited as hindering factors to fertility intentions. Reported hindering imaginaries refer to expected parental duties and the fulfillment of personal aspirations outside of the family. Table 2 displays these categories along with the respondents' exemplary quotations. The most common categories which emerged were the desire for self-realization and freedom as obstacles to deciding to have a(nother) child. The respondents believed that having a(nother) child would not only reduce their time for self-realization and travel, but also limit time for couple activities or career developments. This argument – which accords with SDT theory – contrasts the presence of children with self-fulfillment and champions the latter.

**Table 2:** Imaginaries hindering fertility intentions.

**Categories**	**Exemplary quotations**	**N**
**Desire for self-realization and freedom**		24
Time for self-realization	I would need a little ‘breathing space' because between work and family commitments, I have very little time left for me (male, 36).	
Desire to travel	I am still young and having a child would limit my ability to travel or go out. On the one hand, I would like to have a child, but on the other, I have much more freedom (male, 27).	
Time for couple	You no longer do any couple activities but are always just focused on the children (female, 33).	
Career	Professional growth and fulfillment in my work are currently more important personal aspects than having a child. I don't feel ready at the moment to dedicate myself to raising a child at the same time as my professional growth (female, 31).	
Desire to get married	You must first get married (male, 36).	
**Child requires responsibility and care**		11
Responsibility	The terror of not being able to cope with the responsibility that having more than one child requires (female, 24).	
Care	I already have two little daughters and have no plans to have another. Having children is relatively easy and so is putting a plate of pasta on the table. The problem is raising them, educating them – all of which involves sacrifices for us as parents. It's a job for all intents and purposes, and it's a permanent job (female, 34).	
Engagement	My biggest commitment would be to make him happy not to make his life a roulette wheel (male, 49).	
**Inadequate standard of living for children**		8
Everything they deserve	I don't want to then not be able to afford to raise him in a situation where he can't get what he deserves (male, 33).	
Economic standard	Fear of not being able to provide an adequate economic situation for both children (female, 33).	
**Inadequate personal maturity**	I don't feel that either I or my partner can have any because we are not yet mature, to have a child you have to have a degree of maturity that neither of us has (female, 27).	4

Coding categories and exemplary quotations (*N* = 47).

The desire for self-realization and freedom was not an exclusive feature of highly-educated, career-oriented respondents, but was also observed in those with lower levels of education (Table 1, columns 6 and 7). Moreover, the desire for self-realization as an obstacle to fertility intentions almost disappeared after the arrival of the first child (Table 1, columns 12, 13, and 14). Despite the expectation that the presence of one child should reduce the time for personal activities and increase fears of further reductions in the case of an additional one, this fear decreases in importance after the first child. As with the presence of economic obstacles across parities, this trend seems counterintuitive: The experience of parenthood shapes personal perceptions that directly contrast the objective condition and its subjective perception.

The second most common group of categories was imaginaries of intensive parenting (Hays, 1996; Gauthier et al., 2020). Those imaginaries view the presence of children as an enormous responsibility due to the vast quantities of time and effort required to nurture and educate them. Childrearing is often referred to as a “full-time job” that requires enormous amounts of engagement. These frightened imaginaries of parenthood may also manifest themselves as guilt over not being able to provide “everything a child deserves”, both in economic and non-economic terms. Finally, for some respondents, these feelings of inadequacy were not limited to certain life standards, but also to their whole personal identities, where they spoke of “inadequate personal maturity.”

Certain meso-level conversion factors also emerged. They mostly referred to the presence of other children and the characteristics of their relationships with their partners. The partner characteristics reported as obstacles to fertility intentions referred mostly to the partner's job (specifically regarding unemployment or difficult work-family conciliation). “The current state of my relationship and the work situation of my partner, who is often away on business. Before having a child, I want to make sure I can create the best possible family environment around him or her” (male, 34).

Despite the fact that the open question analyzed thus far concerned personal situational factors, some respondents reported macro-level factors. However, we were able to more accurately analyse macro-economic hindering factors with the answers to the open question on macro-economic aspects.

### Macro-economic hindering factors to fertility intentions

Table 3 shows the codes and frequencies of the macro-economic hindering factors to fertility intentions for the unachieved capability group. The most common group of factors related to economic issues, while the second concerned (the lack of) policies. The respondents cited a general feeling of insecurity and distrust over the country's future economic situation as an obstacle to fertility plans. “Currently, in my country, the economic situation is definitely devastating. There are no positive aspects today to push a couple to have children. Unfortunately, if you are lucky, you can find a temporary job that often – because of the “system” – is not renewed and then you have to start from zero” (female, 26). Regarding economics, the most reported factors were low incomes, precarious jobs or the lack of job opportunities, and generic economic uncertainty (Table 3, columns 1–4). Moreover, the respondents also reported feeling a progressive deterioration of working conditions and rising levels of insecurity. “Everything, there is no more security in anything, nor on the workplace and then on an income, nor at the level of aid from the state, the situation is becoming drastic to say the least” (male, 33). Precarious labor conditions also prohibit people from being granted a mortgage, which is a situation that can be overcome only with the support of affluent families of origin. “The progressive reduction of the most basic social rights: It is now impossible to find a decent permanent job, to obtain a mortgage, to have even a minimum economic security if one does not already come from a wealthy family” (female, 29). In terms of the distribution of the macro-economic hindering factors across different parities (Table 3, columns 5–8), there was a similar distribution of the economic factors related to the personal situation described above (Table 1, columns 11–14). We found that the salience of macro-economic hindering factors decreased with the rise of the number of children.

**Table 3:** Codes and subcodes frequencies of the macro-economic hindering factors to fertility intentions for the unachieved fertility capability group with distribution among different parities.

	**Sample**				**Parity**			
					**0**	**1**	**2**	**3**
**Hindering factors**	**%**	***N***	**%**	***N***	**%**	**%**	**%**	**%**
	**1**	**2**	**3**	**4**	**5**	**6**	**7**	**8**
*Economic*	44	73			48.1	41.5	33.3	50
Low salaries/income			16.3	27	16.5	15.1	16.7	20
Precarious jobs			12	20	12.7	9.4	8.3	30
Generic economic troubles/uncertainty			9.6	16	12.7	9.4	4.2	
Job opportunities (lack of)			8.4	14	11.4	3.8	8.3	10
Growing unemployment			4.8	8	5.1	5.7		10
Women labor discrimination			1.8	3	1.3	3.8		
Cost of children			0.6	1	1.3			
Unemployment subsidies (lack of)			0.6	1		1.9		
*Policies*	38.6	64			36.7	35.8	50	40
Incentives and family policies (lack of)			27.7	46	30.4	20.8	33.3	30
Work-family conciliation policies (lack of)			12.7	21	7.6	18.9	16.7	10
Day-care centers (lack or high cost of)			6.6	11	5.1	9.4	8.3	
Housing policies			1.2	2		1.9	4.2	
*Decaying country - lack of prospects - general insecurity*	4.2	7			5.1	5.7		
*Education opportunities (lack of)*	2.4	4			2.5		4.2	10
*Other*	3	5			5.1		4.2	
*None*	15.1	25			10.1	20.8	16.7	20
*N* of respondents	166				79	53	24	10
% of the sample	100				47.6	31.9	14.5	6

Sample: respondents with fertility intentions < children imaginary [*N* = 166, uncoded document = 26 (15,7%); column % among the coded documents].

The lack of family policies and childcare services was cited by over one-third of the respondents. They referred both to the need for economic support for couples with children and the high cost, or complete lack of, childcare services. “The lack of services to families. The lack or high cost of day-care centers. Poor economic support for families and high taxes that have a very negative impact on family income” (male, 34). The references to lack of family policies were equally distributed among different parities, with a slight increase among respondents with two children (Table 3, columns 5–8). Some respondents reported how family policies are still dependent on the support of the family of origin. Indeed, as one respondent remarked: “Certainly, the subsidies for families in our country are not adequate to the needs. The real welfare is that of the maternal and paternal grandparents, still living and healthy, who give a big hand to the whole family system. Even this morning in order to take this test, one of the two children stayed at their grandparents' house. If I had had to find a babysitter, I would not have taken this test” (male, 31).

As with those of the personal situation, we also performed the analysis of the distribution of the macro-economic hindering factors across different groups of respondents for working conditions, education level, religiosity, and sex. The results (available upon request) showed no particularly different distribution of factors among these different groups.

### Enabling factors to fertility intentions

We analyzed the enabling factors to fertility intentions within the sub-sample of respondents with a high level of fertility intentions (5–10). As with hindering factors, the economic factors were also prominent, but as enabling factors. While a precarious job was regarded as a significant obstacle, the presence of a stable job has the same, though beneficial, salience for positive fertility intentions (Table 4, columns 1 and 2). A stable job was considered necessary as it “enables [one] to support a family” (male, 37). A stable job with a sufficient income level is the economic prerequisite of childbearing. This was reported across different levels of education (Table 4, columns 6–8) and sex, with slightly higher male levels of importance to the presence of a positive economic situation (Table 4, columns 9 and 10).

**Table 4:** Code frequencies of the enabling factors to fertility intentions for the achieved capability group.

**Enabling factors**	**Sample**		**Working condition**			**Education level**			**Religiosity**		**Sex**		**Parity**	
	**%**	***N***	**No work %**	**Temp %**	**Perm %**	**Low %**	**Med %**	**High %**	**Yes %**	**No %**	**F %**	**M %**	**0**	**1**
	1	2	3	4	5	6	7	8	9	10	11	12	13	14
**Micro**														
**Perceptions, Expectations**														
*Economic*	21.6	38	9.4	10	33.8	33.3	25.7	15.9	18.2	20.7	18.3	25.3	22.6	18.2
Stable job	15.8	24	6.3	5	25.7	16.7	17.1	14.3	11.4	15.9	15.5	16	17.2	11.4
Positive economic situation	6.8	11	3.1	5	9.5	25	8.6	1.6	9.1	6.1	2.8	10.7	6.5	6.8
High-income level	2	3			4.1	8.3	1.4	1.6		2.4		4	3.2	
*Age*	15.1	22	9.4	20	14.9	8.3	14.3	17.5	20.5	12.2	15.5	14.7	19.4	9.1
*Housing*	7.5	13	3.1	2.5	12.2	16.7	7.1	6.3	13.6	4.9	7	8	7.5	6.8
*Other*	4.8	8	6.3		6.8	16.7	5.7	1.6	9.1	2.4	4.2	5.3	5.4	4.5
**Imaginaries**	42.9	88	50	40	41.9	50	44.3	41.3	52.3	35.4	43.7	42.7	39.8	38.6
*Children as a means*	23.3	45	34.4	20	20.3	16.7	25.7	22.2	25	19.5	22.5	24	22.6	18.2
*Children as an end*	18.5	30	21.9	17.5	17.6	33.3	11.4	23.8	29.5	12.2	18.3	18.7	15.1	20.5
*Family as an end*	8.2	13	9.4	2.5	10.8	16.7	10	4.8	9.1	8.5	8.5	8	7.5	4.5
**Meso**														
*Partner*														
Relationship	24.7	37	28.1	17.5	27	16.7	30	20.6	15.9	28	31	18.7	30.1	18.2
Fertility intentions	5.5	8		7.5	6.8		7.1	4.8	11.4	3.7		10.7	4.3	4.5
Job	5.5	9	6.3	5	5.4		5.7	6.3	4.5	4.9	8.5	2.7	6.5	2.3
Age	0.7	1			1.4	8.3			2.3			1.3	1.1	
*Family of origin*	11	18	12.5	15	8.1	16.7	14.3	6.3	11.4	9.8	15.5	6.7	10.8	11.4
*Other children: give a sibling*	3.8	6	3.1	5	4.1		5.7	3.2	2.3	2.4	2.8	5.3		13.6
*N* of respondents	146		32	40	74	12	70	63	40	82	71	75	93	44
% of the sample	100		21.9	27.4	50.7	8.2	47.9	43.2	30.1	56.2	48.6	51.4	67.9	32.1

Distributions among different working conditions, education level, sex, and parities (excluded parities of 2 children *N* = 4 and 3 children *N* = 5). Sample: respondents with fertility intentions 5–10 (*N* = 146, uncoded document = 27; column % among the coded documents).

Imaginaries are prominent drivers of fertility intentions. Table 5 reports the different dimensions and exemplary quotations of children and family imaginaries enabling fertility intentions. We classified them into three different groups: Imaginaries of children as a means, children as an end, and family as an end. *Children as a means* was the most reported group of imaginaries. Here, children were viewed as a means toward self-realization, the fulfillment of the couple/family, the joy of nurturing and educating children, a contribution to society, and a means to satisfy grandparents. *Children as an end* refers to children as the greatest joy, something always wanted, or the consequence of love. In the same vein, *family as an end* imaginaries see a family with children as a valuable outcome of the life-course. Both the children and family as an end imaginaries locate family and children as life goals which require no further justification.

**Table 5:** Children and family imaginaries enabling fertility intentions (*N* = 88).

**Categories**	**Exemplary quotations**	**n**
**Children as a means**		**45**
Self (self-realization)	It is one of the things worth living for. A child will make your life happy, fulfilled and inspiring. A beautiful proof of love (male, 44).	
Couple/family (completion of the)	The idea of change and growth. I do not believe in marriage but I have been living with my partner for seven years and the idea of a child would bring to our relationship the growth we need together (female, 35).	
Childcare, education and love for children	The love for my partner and the desire to build something together, and have a child to educate properly (male, 29).	
Society (contribution to)	The desire for social redemption, in a country such as Italy, where everything is against the strengthening of primary social groups such as the family (male, 37).	
Grandparents (satisfaction of)	I would like to give happiness to my family, who want it (female, 38).	
**Children as an end**		**30**
Beautiful/gift/ greatest joy/ worth living	I am blessed to already have two children and I believe there is no greater joy than seeing your children grow up (male, 32).	
Always wanted/desire	Some things start from the heart. Life should be lived with love and every age has its beauty, so becoming a mother is the most beautiful thing in life, the best job there is. It is a value that starts from the deep of the soul and I have always had the sense of becoming a mother since I was little. I am always surrounded by children because I do volunteer work and teach catechism (female, 29).	
Love for children	We both adore children and often fantasize about what it would be like to have one of our own. At the base of my answer there is definitely love (female, 26).	
**Family as an end**		**13**
Numerous	My family to date is composed of me, my partner and my son, and my personal desire to have a large family, living with serenity, joy and respect, and all the values in which I firmly believe (male, 26).	
Value	Optimism in life. We are Catholics and believe in the value of family (male, 28).	
Build a family	My age, being good with my partner of 10 years, the wish to build a family with him, to expand our growth together. The desire to see in our son our true union (female, 32).	
Love for the family	I already have three children, I love the family. Often, it's not easy to reconcile everything, work, home, and children, but when I'm tired in the evening and I go into my children's rooms to say good night, everything passes immediately! So, I would be happy to have another child, but it's not easy! (female, 40).	

Enabling imaginaries were distributed across different working conditions and education levels, with a slight predominance among the unemployed (Table 4, column 3), or less educated respondents (Table 4, column 6). Imaginaries were more often reported by religious than irreligious respondents (Table 4, columns 9 and 10), but they were differentiated by sex (Table 4, columns 11 and 12), or parity (Table 4, columns 13 and 14).

The quality of the relationship with the partner was the most reported meso-level enabling factor. “The serene and stable relationship I have with my partner” (female, 29). This factor was reported mostly by irreligious respondents, women, and the childless (Table 4, columns 9–14). The relationship with the partner and the children as an end imaginaries were more frequently reported by irreligious and religious respondents, respectively. One possible interpretation of this difference is that irreligious respondents afforded a greater salience to the quality of family relationships due to the absence of religious family goals. The partner's fertility intentions as enabling factors were reported only by men (Table 4 columns 11 and 12), while the support of the family of origin was reported more often by women (Table 4 columns 13 and 14).

We searched for the macro-economic enabling factors for fertility intentions in the answers to the related open question for the achieved capability group of respondents. However, we found no evidence of such country macro-economic enabling factors. Indeed, all the reported macro-economic factors were considered as obstacles.

## Discussion

**Figure 2:**
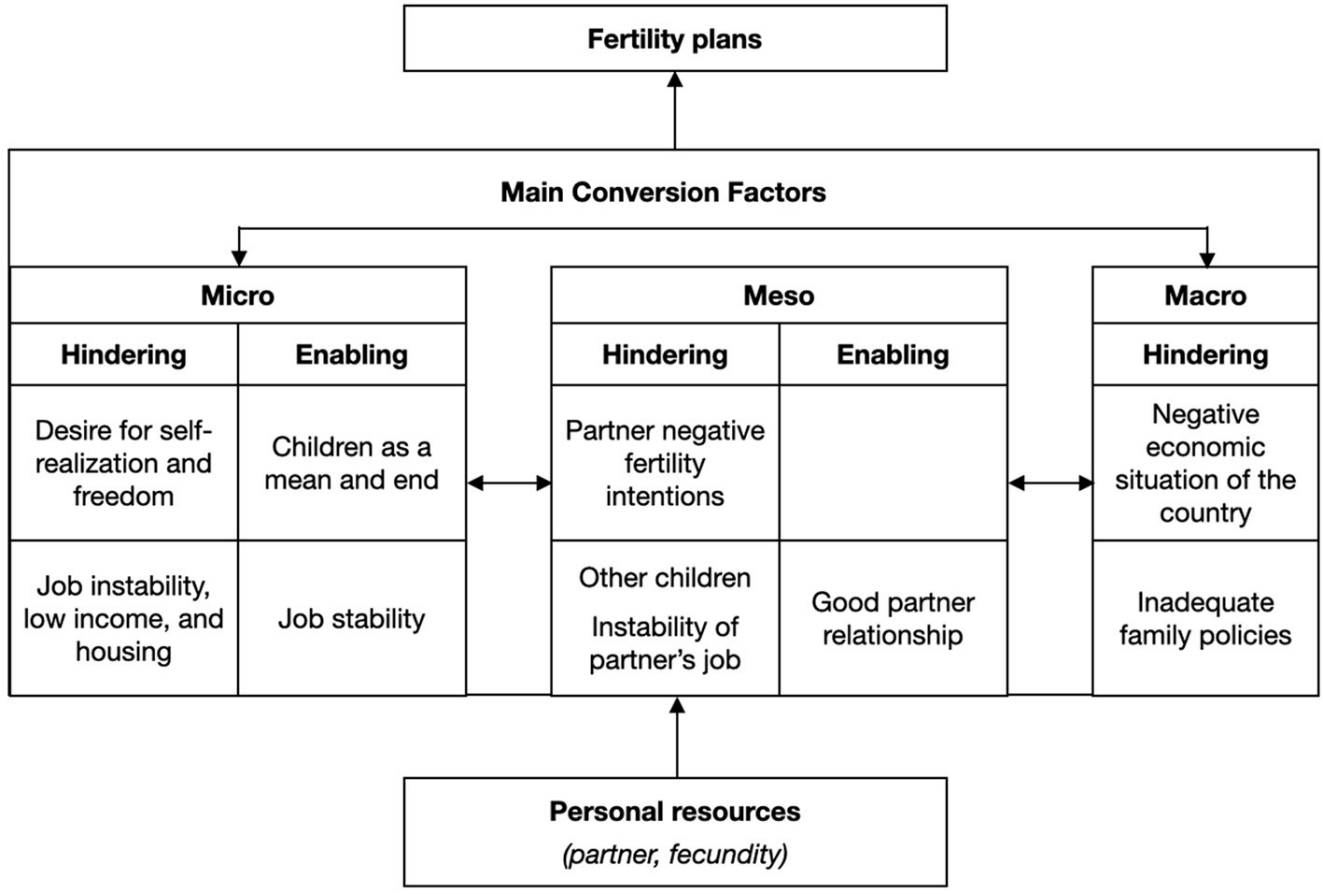
The main conversion factors of fertility agency.

The results show that economic factors and imaginaries are highly prominent in enabling/hindering fertility agency. Figure 2 synthesizes the main conversion factors of fertility agency. Most reported factors belong to the micro-level, and it is interesting to note how personal imaginaries and economic factors simultaneously hinder and enable fertility plans.

Our inductive approach using open-ended questions allowed us to describe enabling and hindering family imaginaries (Boyatzis, 1998). Hindering imaginaries associated children with the loss of free time, and opportunities for career and self-development. These imaginaries thus support SDT theory, which states that individuals have now reprioritised their careers and self-actualisation over family and childbearing (Van de Kaa, 1987; Lesthaeghe, 2020). However, the relationship between career and family plans is not straightforward, and may instead elicit both a substitution and spill-over effect (Huinink and Kohli, 2014, p. 1296–7). Moreover, having children is an enormous responsibility that requires high levels of engagement and time, as per the intensive parenting and total motherhood models (Hays, 1996; Gauthier et al., 2020; Lebano and Jamieson, 2020). However, while in traditional gender roles the care of children is mostly assigned to the mother, we found intensive parenting imaginaries also spread among our male respondents.

Enabling children imaginaries describe children and the family as the “greatest joy” that overcome the difficulties that may result from parenthood. Children and family, however, are both considered as an “end” and as a “means.” Interestingly, children as a means imaginaries suggest that children may also lead to self-fulfillment (Arendell, 2000) through their care and education: A trajectory of self-realization underestimated by SDT theory. Remaining within SDT theory, van de Kaa (2001, p. 320) noted that having children may “constitute an important element in their [men and women] perception of wellbeing and self-realization”. The exact meaning of this imagined self-realization has remained largely unexplored. Enabling imaginaries confirm the idea of raising children as a personal project (Wall, 2010), but suggest a rich variety of children and family imaginaries behind the two-child ideal family size. We found that enabling children and family imaginaries were equally distributed across different working conditions, education level, sex, and parities, albeit with a slight increase among the less educated and unemployed. This result is in line with recent research on future making and class differences that challenge the assumption that disinterest in higher education reflects low aspirations and narrow time horizons rather than alternative future plans involving family and community ties. According to Lund, the traditional assumptions on future making and class differences often reflect “an inherent bourgeois gaze within sociology that shapes sociologists' interest in the valuation of how time is used and, hence, the social hierarchization of outcomes” (Lund, 2018, p. 562). For the more vulnerable respondents, parenthood was occasionally considered a path toward self-realization that could be achieved more easily than, say, professional success. This pattern accords with the uncertainty reduction framework (Friedman et al., 1994). The variety of enabling children imaginaries contrasts the idea of the family as a “zombie category” (Beck, 2002, p. 204), and contributes to children being more frequently viewed as conduits for self-fulfillment. Both the hindering and enabling imaginaries are crucial anchors for the hyperprojective capacity of fertility plans, in that they can elicit a desired/frightening future able to guide present decisions and life-course plans. The described dimensions of family and children imaginaries may be useful for developing comparative studies and dedicated surveys. The centrality of personal family imaginaries is one of the main drivers of fertility plans that emerged from the narratives. This challenges some of the literature's assumptions on fertility plans regarding the dominant role of economic and structural factors, and the assumed determinism between personal resources and fertility agency (see also Vignoli et al., 2020a).

Regarding the economic conversion factors, economic concerns and the widespread feeling of insecurity confirm the proposition that globalization and neoliberal policies have impacted fertility agency capacity (Mills and Blossfeld, 2013). However, the reported narratives suggest avoiding a direct determinism between the objective employment condition and a real fertility agency capacity (Guetto et al., 2020; Vignoli et al., 2020a,b, 2022; Manning et al., 2022): The fear generated from precarious jobs was also cited as a hindering factor by stably-employed respondents. Insecurity spread by uncertain labor conditions also affected the fertility agency of those with an objectively positive employment condition. The Italian macro-economic context provided no enabling factors to fertility intentions: It is depicted only as a threat to fertility plans and stability. The micro-, meso-, and macro-level conversion factors clearly interact with one another (Billari, 2015; Bernardi et al., 2019), although examining their internal dynamics was outside of the scope of this analysis (see Cisotto et al., 2022; Rutigliano and Lozano, 2022).

The distribution of the economic hindering factors among different parities was one of our most unexpected findings. The economic hindering factors referring both to the personal and macro-level situation were more frequently reported by childless respondents than parents, regardless of the income level. This seems to suggest that the experience of parenthood is not a monetary calculus that discounts the increasing cost of a(nother) child from the household income. Parenthood is a life-changing experience that shapes the meaning and salience of different aspects of life and personal identities and, in this case, reduces the importance of economic factors to further fertility plans. These findings seem to contradict the “relative income hypothesis” (Easterlin, 1980) by suggesting a more *situated* evaluation also for the economic prerequisite of fertility. While income is an objective measure of available resources, the exact meaning of its value and appropriateness for parents with children is attributed in view of specific conversion factors of fertility agency, such as personal family imaginaries or the quality of a couple's relationship. This finding confirms the need to avoid a direct determinism between personal resources and fertility agency: The suggested focus on factors which convert resources into achievements seems the most suitable strategy for understanding the emergence of fertility agency.

## Conclusion

Agency is a crucial dimension of a life-course. Without it, life-courses would be reduced to unrecognizable, deterministic patterns, where life-course outcomes would be entirely predetermined by habitus, past experiences, and personality traits (Kohn, 1989). However, this capacity cannot be abridged to personal will and, in the case of such long-term plans as fertility, to a rational cost–benefit calculation. Fertility plans are a prominent case of agency as they allow for hyperprojectivity (Mische, 2014). As opposed to other decisions in an individual's life, such as marriage, labor, and residence, childbearing is likely the last life-course decision in contemporary societies that continues to be perceived as irreversible. It is a crucial turning point because, according to fertility/childless plans, career, housing, and partnership opportunities are often (re)considered in line with the expected needs of children.

Fertility agency can refer both to the effects of the complex interactions between personal and structural resources, as well as to situational factors that shape personal narratives of the future. Fertility plans are often at the crossroads of contrasting possibilities between self-fulfillment and career development, and the two-child ideal family size. Indeed, family preferences are often neither straightforward nor fixed, but instead emerge in the form of *despite* narratives and *within* the occurrence of contrasting possibilities.^12^ In Sen's terms (1992; 2005) (Hvinden and Halvorsen, 2018), fertility agency capability inputs are not directly converted into agency freedom or achievement due to more or less contingent conversion factors that may enable or hinder fertility plans and, consequently, fertility behavior. The proposed framework based on the CA and the temporal oriented account of agency is an open platform with which to elicit specific research questions on fertility agency. The notion of conversion factors in particular seems crucial for disentangling the network of heterogeneous elements that can hinder or enable fertility agency, and allows one to shift focus away from structural factors related to social position and individual psychological characteristics to more situated elements. The notion of situated agency fits more suitably with fertility agency (Peter, 2003; Hobson, 2014, 2018; Choi et al., 2019). Moreover, our framework allows the consideration of different temporal orientations (Emirbayer and Mische, 1998; Bazzani, 2022) of the heterogeneous elements involved in fertility agency: Both the present condition of the respondents and their future expectations and imaginaries emerged as key conversion factors for fertility agency (Vignoli et al., 2020a; for empirical evidence, see Guetto et al., 2022; Manning et al., 2022). The micro-, meso-, and macro-level conversion factors can thus be investigated in their iterative, evaluative, and projective temporal orientations. The study of the conversion factors of projectivity is a new frontier for research (Hobson and Zimmermann, 2022), to the best of our knowledge, this study is the first empirical study aimed to explore this link – at least in the realm of fertility research. This exploratory study allowed us to tap into temporal landscapes of risk and uncertainty (Tavory and Eliasoph, 2013) in terms of both the personal (economic) situations of respondents and the general economic condition of their country. Of course, the identified conversion factors are far from exhaustive. Indeed, further factors could be found by future research following the proposed framework for the study of fertility agency, which could in turn affect different subjects and study contexts.

The study has limitations. First, despite the sample being larger than the norm for qualitative samples, it is not representative, nor is it clear to what extent our results are generalisable beyond this specific group. However, the heterogeneity of the respondents' working conditions, as well as the stratified analysis for working condition, education level, sex, religiosity, and parity provide the results with a broader validity. Second, despite social desirability having been avoided due to the absence of an interviewer, self-reported reasoning is exposed to possible cognitive and motivational biases (Burke and Stets, 1999). However, while a causal analysis may always find a deeper reason behind observed behavior, we sought to describe the conversion factors involved in the fertility decision-making process as accurately as possible. Narratives have a clear motivating power in focusing life-course goals and sustaining the daily efforts their achievement require (Tuckett, 2018; Vignoli et al., 2020a). Third, while open questions allowed for an inductive approach, some unexpected results could have been more accurately interpreted with more qualitative insights. For instance, the different salience levels of economic factors across different parities, or the different imaginaries between religious and non-religious respondents, could have been more comprehensively explored with in-depth interviews with convenient samples. Moreover, the analysis of practical evaluative agency could possibly be more effectively conducted with interviews focusing on the moment when the childbearing decision was (not) taken (Tuckett, 2018). Fourth, the paper is affected by context- and time-specificities. Since labor patterns differ with varying workplace structures, social policies, and cultural norms (Neyer et al., 2013), future research should examine whether our findings translate to other countries. In particular, while the CA has been applied to the study of several research topics in the Global South contexts (Byskov, 2018), and we can expect that our framework could also be fruitfully applied to the study of fertility agency in such contexts, we can speculate that the conversion factors of fertility agency in the Global South would vary from those presented in this research.

The several social dimensions and dynamics that emerged in our results support the consideration of fertility agency as a prominent area of agency research. We sought to help bridge the gap between two pieces of literature that developed mostly separately, but could benefit from interaction. Indeed, demographic research can benefit from agency and future-making frameworks, while fertility plans (clearly a prominent case of agency) deserves greater attention from sociological research on agency. Fertility plans are a huge space of hyperprojectivity capable of shaping entire life-courses and transforming daily life into a long-term binding process. Despite the fact that the outcomes of fertility plans and parenthood can never be consistently predicted with accuracy, their narratives may help stabilize uncertainties, orient decisions in several life domains, and provide reasons for the enduring efforts of fertility agency.

## Data availability statement

The datasets presented in this article are not readily available because Identifiable data. Requests to access the datasets should be directed to GB, giacomo.bazzani@unifi.it.

## Ethics statement

The studies involving human participants were reviewed and approved by the University of Florence. The patients/participants provided their written informed consent to participate in this study.

## Author contributions

All authors listed have made a substantial, direct, and intellectual contribution to the work and approved it for publication.

## Funding

This work was supported by the European Research Council (ERC) under the European Union's Horizon 2020 research and innovation programme, grant agreement n° DLV-72596 under the EU-FER project (PI: DV).

## Conflict of interest

The authors declare that the research was conducted in the absence of any commercial or financial relationships that could be construed as a potential conflict of interest.

## Publisher's note

All claims expressed in this article are solely those of the authors and do not necessarily represent those of their affiliated organizations, or those of the publisher, the editors and the reviewers. Any product that may be evaluated in this article, or claim that may be made by its manufacturer, is not guaranteed or endorsed by the publisher.

## Footnotes

^1^Sen defines an agent as “someone who acts and brings about change, and whose achievements can be judged in terms of her own values and objectives, whether or not we assess them in terms of some external criteria as well” (Sen, 1999, p. 19).

^2^The gap between “agency freedom” and “agency achievements” is a crucial dimension for the sociological understanding of agency dynamics. The former is “one's freedom to bring about the achievements one values and attempts to produce” (Sen, 1992, p. 57), while the latter is “the realization of goals one has reason to pursue” (ibid.: 56; see also Gangas, 2016).

^3^Agency is a complex social process whose outcomes cannot be easily deduced from past observations due to its being “a temporally embedded process of social engagement, informed by the past (in its habitual aspect), but also oriented toward the future (as a capacity to imagine alternative possibilities) and toward the present (as a capacity to contextualize past habits and future projects within the contingencies of the moment)” (Emirbayer and Mische, 1998, p. 963).

^4^In the risk condition, the probability distribution of different outcomes can be estimated, whereas decisions are guided by routines or narratives of the future that can be somewhat plausible and normatively oriented in the uncertainty condition (Tavory and Eliasoph, 2013; Beckert, 2016; Tuckett and Nikolic, 2017).

^5^In this situation, “the objects experienced in following out a course of action,” that were previously unnoticed, start to “attract, repel, satisfy, annoy, promote and retard” (Dewey, 1930, p. 192).

^6^“The activation and expression of competing attitudes, the cognitive structures to which they are linked, and the behaviors that follow from them are elicited by social contexts” (DiMaggio, 2014, p. 233–234).

^7^A previous attempt to identify conversion factors of fertility intentions can be seen in Fahlén (2013). However, it is a framework that mostly considers family policies and employment position.

^8^The typology of the conversion factors cannot be exhaustive of all the elements that may occur in the conversion processes in every specific situation. However, they may still be useful in guiding researchers toward suggesting some possible elements involved and eliciting specific research questions. Some elements of the schema, such as personality traits or institutions, may be considered both resources or conversion factors, depending on the specific research question. Indeed, resources and conversion factors cannot be distinguished by their nature, but rather for the role played in the specific agency dynamic (Zimmermann, 2006; Hobson, 2014). This is “a question of the researcher's perspective to judge what type of relevant variance (conversion factors) needs to be considered within the same endowment of personal and structural “sources of capability input”” (Hvinden and Halvorsen, 2018, p. 874). However, the conversion factors generally represent the situational variance that mediates the chance that the available personal and structural resources belonging to the same target group are converted into real agency freedom or achievements (Bazzani, 2022). The schema provides a meta-theory of agency, useful for eliciting more specific research questions on agency dynamics.

^9^Respondents with a migratory background were too small in number to allow a separate analysis.

^10^The limited size of the subgroups of respondents did not allow for a precise estimation of the differences in the codes' distribution. However, we considered differences in the distribution of the same code among different groups of respondents when the gap exceeded 10%.

^11^Monthly salary (€ mean): childless = 1,180, one child = 1,206, two children = 1,328. Considering the Italian fiscal system's tax deductions for dependent children, the gross salary was highly similar across the different groups of respondents.

^12^In this sense, Duncan talks about family agency as a ‘bricolage process' (Duncan, 2011; Carter and Duncan, 2017).
