# Supplementary material for: The agency of fertility plans
Source: Front Sociol. 2022 Nov 25;7:923756. doi: 10.3389/fsoc.2022.923756 (PMC9732582; doi:10.3389/fsoc.2022.923756)
Supplement: Supplementary file 1 [file Table_1.DOCX]

Supplementary Material

**Appendix**

Table A1. Sample and subsamples descriptive statistics

|  | **Unachieved capability** | | | **Entitled** | | | **Sample** | | |
| --- | --- | --- | --- | --- | --- | --- | --- | --- | --- |
|  | Mean | SD | N | Mean | SD | N | Mean | SD | N |
| **Fertility intentions** | 3.31 | 2.8 | 166 | 8.08 | 1.84 | 146 | 4.90 | 3.76 | 276 |
| 0 | .22 |  | 36 |  |  |  | .19 |  | 53 |
| 1 | .17 |  | 28 |  |  |  | .11 |  | 30 |
| 2 | .08 |  | 14 |  |  |  | .06 |  | 16 |
| 3 | .07 |  | 12 |  |  |  | .05 |  | 13 |
| 4 | .1 |  | 17 |  |  |  | .07 |  | 18 |
| 5 | .09 |  | 15 | .13 |  | 19 | .07 |  | 19 |
| 6 | .07 |  | 11 | .12 |  | 17 | .06 |  | 17 |
| 7 | .11 |  | 19 | .15 |  | 22 | .08 |  | 22 |
| 8 | .07 |  | 11 | .13 |  | 20 | .07 |  | 20 |
| 9 | .02 |  | 3 | .8 |  | 12 | .04 |  | 12 |
| 10 |  |  |  | .38 |  | 56 | .2 |  | 56 |
|  |  |  |  |  |  |  |  |  |  |
| **Children imaginary** | 7.10 | 2.75 | 166 | 8.62 | 1.84 | 146 | 6.94 | 3.22 | 276 |
| 0 |  |  |  | .01 |  | 2 | .07 |  | 19 |
| 1 | .02 |  | 4 |  |  |  | .03 |  | 8 |
| 2 | .07 |  | 12 |  |  |  | .05 |  | 14 |
| 3 | .06 |  | 10 | .01 |  | 1 | .04 |  | 11 |
| 4 | .05 |  | 8 | .01 |  | 1 | .03 |  | 9 |
| 5 | .07 |  | 11 | .04 |  | 6 | .06 |  | 17 |
| 6 | .08 |  | 14 | .03 |  | 5 | .07 |  | 18 |
| 7 | .12 |  | 20 | .11 |  | 16 | .11 |  | 29 |
| 8 | .13 |  | 21 | .16 |  | 23 | .12 |  | 32 |
| 9 | .11 |  | 19 | .17 |  | 25 | .11 |  | 31 |
| 10 | .28 |  | 47 | .46 |  | 67 | .32 |  | 88 |
|  |  |  |  |  |  |  |  |  |  |
| **Education level** |  |  | 166 |  |  | 146 |  |  | 276 |
| *High* | .30 |  | 50 | .43 |  | 63 | .51 |  | 102 |
| *Medium* | .56 |  | 93 | .48 |  | 12 | .37 |  | 141 |
| *Low* | .14 |  | 22 | .09 |  | 70 | .12 |  | 32 |
| *Missing* |  |  | 1 |  |  | 1 |  |  | 1 |
|  |  |  |  |  |  |  |  |  |  |
| **Working condition** |  |  | 166 |  |  | 146 |  |  | 276 |
| *Jobless* | .28 |  | 46 | .22 |  | 32 | .26 |  | 72 |
| *Permanent job* | .42 |  | 70 | .51 |  | 74 | .45 |  | 125 |
| *Temporary job* | .30 |  | 50 | .27 |  | 40 | .29 |  | 79 |
|  |  |  |  |  |  |  |  |  |  |
| **Female** | .50 |  | 83 | .49 |  | 71 | .49 |  | 135 |

Table A2. Economic hindering factors among different parities

|  | **Childless** | **1 Child** | **2 Children** | **3 Children** |
| --- | --- | --- | --- | --- |
| **Precarious job** | In the historical period in which we find ourselves, we young people with no job security and difficulties in reaching a stable position, can hardly set ourselves such goals. We need to think more about surviving in the current world of work than to think about having a child. (Male 34) | I'd rather wait for a better setup and a more stable job to proceed with the second one. (Male 30) | The presence of a precarious job. (Female 33) | I work a precarious job, as does my husband. (Female 37) |
| **Low income** | The main problems are the cost of living. (Female 27) | The economic situation, already having a child aged 6, you would see a doubling of costs and family expenses. (Male 38) | The salary I get is too low to support a large family. (Male 43) | The choice was determined by the fact that I already have three children, and having a fourth would be an enormous commitment, both economically and organisationally. (Female 37) |
